# Repeatedly Northwards and Upwards: Southern African Grasslands Fuel the Colonization of the African Sky Islands in *Helichrysum* (Compositae)

**DOI:** 10.3390/plants12112213

**Published:** 2023-06-03

**Authors:** Carme Blanco-Gavaldà, Mercè Galbany-Casals, Alfonso Susanna, Santiago Andrés-Sánchez, Randall J. Bayer, Christian Brochmann, Glynis V. Cron, Nicola G. Bergh, Núria Garcia-Jacas, Abel Gizaw, Martha Kandziora, Filip Kolář, Javier López-Alvarado, Frederik Leliaert, Rokiman Letsara, Lucía D. Moreyra, Sylvain G. Razafimandimbison, Roswitha Schmickl, Cristina Roquet

**Affiliations:** 1 Systematics and Evolution of Vascular Plants—Associated Unit to CSIC by IBB, Department of Animal Biology, Plant Biology and Ecology, Faculty of Biosciences, Autonomous University of Barcelona, ES-08193 Bellaterra, Spain; 2 Botanic Institute of Barcelona (IBB), CSIC-Ajuntament de Barcelona, Pg. Migdia s/n, ES-08038 Barcelona, Spain; 3 Department of Botany and Plant Physiology and Plant DNA Biobank, DNA National Bank, University of Salamanca, Edificio I+D+i, Espejo St., ES-37007 Salamanca, Spain; 4 Department of Biological Sciences, Center for Biodiversity, University of Memphis, Memphis, TN 38152, USA; 5 Natural History Museum, University of Oslo, P.O. Box 1172, NO-0318 Oslo, Norway; 6 School of Animal, Plant and Environmental Sciences, University of the Witwatersrand, Private Bag 3, Johannesburg 2050, South Africa; 7 Foundational Biodiversity Science, Kirstenbosch Research Centre, South African National Biodiversity Institute, Private Bag X7, Newlands, Cape Town 7735, South Africa; 8 Department of Plant Biology and Biodiversity Management, Addis Ababa University, Addis Ababa P.O. Box 3434, Ethiopia; 9 Department of Botany, Faculty of Science, Charles University in Prague, Benátská 2, CZ-12801 Prague, Czech Republic; 10 Institute of Botany, Academy of Sciences of the Czech Republic, CZ-25243 Průhonice, Czech Republic; 11 Meise Botanic Garden, Nieuwelaan 38, BE-1860 Meise, Belgium; 12 Herbarium of the Parc Botanique et Zoologique of Tsimbazaza (PBZT), Antananarivo 3G9G+V6C, Madagascar; 13 Department of Botany, Swedish Museum of Natural History, P.O. Box 50007, SE-104 05 Stockholm, Sweden

**Keywords:** Afroalpine, Afromontane, biogeography, Asteraceae, long-distance dispersal, evolution, *Helichrysum*, phylogeny, target-enrichment

## Abstract

The Afromontane and Afroalpine areas constitute some of the main biodiversity hotspots of Africa. They are particularly rich in plant endemics, but the biogeographic origins and evolutionary processes leading to this outstanding diversity are poorly understood. We performed phylogenomic and biogeographic analyses of one of the most species-rich plant genera in these mountains, *Helichrysum* (Compositae-Gnaphalieae). Most previous studies have focused on Afroalpine elements of Eurasian origin, and the southern African origin of *Helichrysum* provides an interesting counterexample. We obtained a comprehensive nuclear dataset from 304 species (≈50% of the genus) using target-enrichment with the Compositae1061 probe set. Summary-coalescent and concatenation approaches combined with paralog recovery yielded congruent, well-resolved phylogenies. Ancestral range estimations revealed that *Helichrysum* originated in arid southern Africa, whereas the southern African grasslands were the source of most lineages that dispersed within and outside Africa. Colonization of the tropical Afromontane and Afroalpine areas occurred repeatedly throughout the Miocene–Pliocene. This timing coincides with mountain uplift and the onset of glacial cycles, which together may have facilitated both speciation and intermountain gene flow, contributing to the evolution of the Afroalpine flora.

## 1. Introduction

Some mountain systems are highly isolated from each other by valleys and lowlands with drastically different climatic conditions, and such mountains are commonly referred to as “sky islands” [1]. Clusters of sky islands are similar in several respects to oceanic archipelagos, providing natural replicates for studies of evolutionary and biogeographic processes that have resulted in current biodiversity patterns. A prominent example of sky island archipelagos is found in Africa, where the highest mountains host enigmatic floras with outstanding levels of endemism [2,3,4].

The Afromontane area is one of the biodiversity hotspots of Africa and has long been recognized as an independent floristic region [4,5,6]. It holds ≈4000 vascular plant species, of which ≈3000 are endemic [3]. This area extends from the escarpment mountains of the Yemen to the high massifs of the South African Western Cape, with some outliers such as Mount Cameroon to the west, Jebel Marra to the north, and the highest mountains of Madagascar to the southeast [7,8] (but see Carbutt and Edwards 2015 [9] for a discussion). The formation of many of the highest areas was coupled with the beginning of the mid-to-late Miocene aridification and cooling, which contributed to the development of deserts and semi-desert conditions along the southwestern parts of southern Africa, as well as to the predominance of savannah and subtropical landscapes across large parts of the continent. At present, these mountains constitute temperate and humid refugia, often isolated from each other by vast expanses with markedly different environmental conditions, such as drier and warmer lowlands [10]. Despite their current isolation, patches of montane forest in some of these elevated areas may have been connected during interglacial periods [5,11]. There are only a few studies focusing on the evolution of strictly Afromontane lineages (e.g., Mairal et al., 2015 [12]).

In contrast to the Afromontane flora, the Afroalpine flora has received much more attention. The Afroalpine area comprises the upper part of the highest mountains of Africa’s mainland, embedded within the Afromontane area. It occurs above the tree line, usually at elevations around 3200 m above sea level (m.a.s.l.) and is characterized by alpine grasslands with gigantic *Dendrosenecio* (Hauman) B. Nord. and *Lobelia* Mill., *Alchemilla* L. shrublands, and open communities with *Erica* L. and *Helichrysum* Mill. [4]. Afroalpine plants are adapted to an environment with extreme diurnal temperature variation (warm days and frosty nights), high insolation, and less precipitation than in Afromontane areas [13]. The Afroalpine flora harbors only 520 plant species but shows one of the highest proportions of endemism in the world, possibly due to the interplay of small areas and extreme isolation [14]. Many species are, however, shared between massifs; single-mountain endemics are rare, representing only 15% of the flora [4].

It has long been recognized that the tropical Afromontane and Afroalpine areas have strong phytogeographic affinities to temperate areas in other parts of the world [5,15,16]. A two-way migration-dispersal route connecting Eurasia and South Africa via the highest mountains of Yemen and the Drakensberg highlands of eastern South Africa, through the mountain systems of eastern Africa, has been proposed [16,17,18,19] and confirmed in many phylogenetic studies (e.g., *Alchemilla*, *Carex* L., *Ranunculus* L. [20]). Concerning the assembly of the Afroalpine flora, it seems that immigration of cold-adapted lineages from remote temperate regions has been more frequent than immigration of lineages from the surrounding tropical regions, which necessitates niche shifts [14,20]. Long-distance dispersal (LDD) appears to be the main driver shaping the current floristic and genetic diversity of the Afroalpine flora [14,20]. Nevertheless, gradual expansion during glacial times may have been possible through corridors of grasslands or open forests for drought-tolerant Afroalpine species, or for Afroalpine species with low elevation boundaries [21], followed by contraction into refugia during warmer periods [22].

Colonization of tropical Afromontane and Afroalpine areas from the south following the aforementioned dispersal route has been reported for some plant groups [18,23]. However, only a few studies have focused on this, yielding poorly resolved phylogenies (e.g., Galbany-Casals et al., 2014 [24]). Due to the lack of phylogenetic resolution, it is currently unclear whether such Afroalpine lineages originated in southern Africa and dispersed towards the north through LDD from the highest areas of the Drakensberg Mountains, which may have constituted a stepping-stone area [22], or whether they colonized the tropical Afroalpine area after expanding to the surrounding tropical lowlands and tropical Afromontane areas [25].

**Figure 1:**
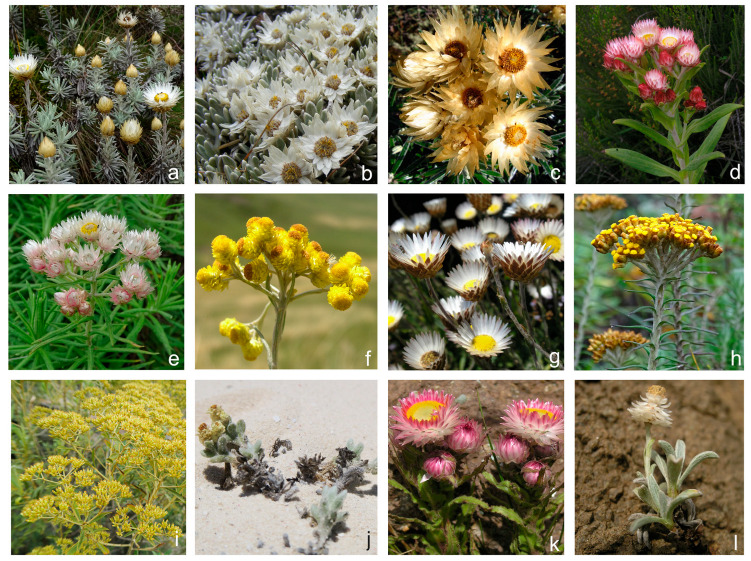
Species of *Helichrysum* illustrating the wide variety of morphologies present in the genus (Photos: Mercè Galbany-Casals; (**b**): Filip Kolář; (**c**): Cristina Roquet): (**a**) *H. newii* Oliv. & Hiern (tropical Afroalpine; Aberdare Mts., Mt. Elgon, Mt. Kenya, Mt. Kilimanjaro, Mt. Meru, Virunga Mts.); (**b**) *H. gofense* Cufod (tropical Afroalpine; Bale Mts.); (**c**) *H. stuhlmannii* O. Hoffm. (tropical Afroalpine; Rwenzori Mts.); (**d**) *H. formosissimum* Sch. Bip. (widely distributed across tropical Afromontane areas); (**e**) *H. argyranthum* O. Hoffm. (widely distributed across tropical Afromontane areas); (**f**) *H. splendidum* Less. (southern African grasslands, and widely distributed across tropical Afromontane and Afroalpine areas); (**g**) *H. confertifolium* Klatt (southern African grasslands); (**h**) *H. helothamnus* Moeser (tropical Afromontane); (**i**) *H. gymnocephalum* Humbert (Madagascar); (**j**) *H. mahafaly* Humbert (Madagascar); (**k**) *H. elegantissimum* DC. (southern African grasslands); (**l**) *H. chionosphaerum* DC. (southern African grasslands).

The Compositae is the most diverse plant family in the tropical Afromontane and Afroalpine areas, and *Helichrysum* (Gnaphalieae) is one of the most species-rich genera, with ≈60 tropical Afromontane and 12 strictly tropical Afroalpine species. The genus is considered an iconic component of these floras, dominating many plant communities, and often having beautiful and showy capitula (Figure 1). Globally, the genus comprises around 500–600 species in total, with its highest species diversity found in Africa (≈314 species [25,26,27,28]), where more than half of the species are montane. Southern African mountains harbor 132 species, of which 24 are endemic to highest part of the Drakensberg mountains, called the Drakensberg Mountain Centre [29], while others grow in the southern African grasslands and some extend into tropical Afromontane areas. The other species are found in various ecosystems, including savannahs, shrublands in the Cape Region, deserts, semi-deserts, and humid coastal forests. In Madagascar, there are 115 species of *Helichrysum*, almost all of them endemic, and a considerable proportion (44%) occupy montane habitats [30].

Previous studies suggested that the genus *Helichrysum* originated in western South Africa during the early to middle Miocene, with subsequent northward migrations to the rest of Africa including Madagascar, as well as to Eurasia [24,28,31]. These studies hypothesized that several independent colonizations of the tropical Afromontane and Afroalpine areas occurred along a dispersal route through the Drakensberg range. The closest relatives of the tropical Afroalpine lineages of *Helichrysum* were inferred to be of tropical Afromontane, Malagasy, and southern African origin [24,31], not Eurasian as it is the most frequent scenario for tropical Afroalpine groups (see examples in Brochmann et al., 2021 [20]). However, these hypotheses were based on small taxonomic datasets and phylogenies that were poorly resolved, likely because they were based on sequences of few DNA markers, i.e., the largest *Helichrysum* phylogeny included 145 species, representing around 25% of the genus, and was based only in two nuclear DNA markers (ITS and ETS) [24].

Our main goal is to investigate the evolutionary and biogeographic history of the genus *Helichrysum*, as an example of a plant group with southern African origins that colonized the tropical Afromontane and Afroalpine areas, in order to contribute to the understanding of the origins of the enigmatic Afromontane and Afroalpine floras. To overcome the taxonomic and geographic sampling limitations of previous studies of *Helichrysum*, we obtained samples of 304 species (representing 50% of the genus) with emphasis on the African species, and generated sequences of nuclear loci using the target-enrichment approach [32] with the Compositae1061 probe set [33]. Moreover, we applied a methodological approach that accounts for paralogy to infer phylogenetic relationships [34], which we used to determine the geographic and temporal origin of the tropical Afromontane and Afroalpine *Helichrysum*. Our specific aims were to determine (1) whether tropical Afromontane and Afroalpine lineages originated from a single or multiple colonization events; (2) whether they originated from geographically close tropical lowland ancestors, which would imply a niche shift to tolerate cold in the case of Afromontane species and frost in the case of Afroalpine species, or from frost (cold)-tolerant temperate lineages that reached tropical Afroalpine mountains through LDD; and (3) in which geological and climatic context the main biogeographic processes took place.

## 2. Materials and Methods

### 2.1. Taxon Sampling

We sampled approximately 50% of the genus *Helichrysum*, including 304 species from all taxonomic groups and geographic areas, which doubles the number of species included in the largest nuclear phylogeny of the genus published up to now [24]. We also included taxa from smaller genera nested within the *Helichrysum–Anaphalis–Pseudognaphalium* (HAP) clade [24,35]: *Achyrocline* Less. (five species), *Anaphalis* DC. (nine species), *Pseudognaphalium* Kirp. (fifteen species), *Syncephalum* DC. (one species), and the monospecific genus *Humeocline* Anderb. We chose *Syncarpha staehelina* (L.) B. Nord. as the outgroup based on previous phylogenetic results at the tribal level [35,36].

When possible, samples were collected in the field and dried in silica-gel by team members or collaborators, and a voucher of each was deposited in the correspondent herbaria. For species that we could not collect in the field, we sampled herbarium material with the authorization of curators from various herbaria: BC, BCN, BNRH, BR, CANB, CONC, E, LP, MA, MADJ, MEXU, MO, NBG, O, P, PRE, RSA, S, SALA, SI, US, and W (acronyms from Thiers [37]; studied materials in Table S1). The identities of field-collected and herbarium-sampled specimens were determined and checked by reference to taxonomic literature [26,30,38,39,40,41,42,43,44,45,46,47,48,49,50,51,52], with type images from JSTOR Global Plants online herbarium databases and specimens at the herbaria listed above. We also added to our dataset the raw sequence reads of 21 specimens previously generated for the Czech Science Foundation GAČR project No. 20-10878S.

### 2.2. DNA Extraction, Library Preparation, Target Capture, and Sequencing

Between 10 and 30 mg of dried plant material per sample was homogenized with a Mixer Mill MM 301 (Retsch, Haan, Germany). DNA extractions were performed using the E.N.Z.A^®^ SP Plant DNA Kit (Omega Bio-Tek Inc., Norcross, GA, USA) following the manufacturer’s instructions. Extracted DNA was sent in parafilm-sealed 1.5 mL tubes to Daicel Arbor Biosciences (Ann Arbor, MI, USA), where library preparation, target enrichment, and sequencing was performed as follows: Total DNA was quantified using an intercalating fluorescent dye (Quant-iT PicoGreen dsDNA Assay Kit, Thermo Scientific, Waltham, MA, USA). DNA quality of a subset of samples was assessed via Agilent Bioanalyzer (Agilent Technologies, Santa Clara, CA, USA). A total of 80% of the available mass up to 4 μg was brought to 100 μL with nuclease-free water and taken through a sonication and size-selection protocol targeting an average insert length of 500 bp. Next, up to 200 ng DNA was used in a library preparation method optimized for target enrichment. Unique dual-index combinations were added to each sample via 6–8 cycles of PCR amplification. The indexed libraries were quantified with both a spectrofluorimetric assay and a quantitative PCR assay. Prior to enrichment, up to 125 ng of each library, was pooled (10-plex reactions) and dried down to 7 µL by vacuum centrifugation.

Target enrichment was performed following the myBaits v.5 protocol with an overnight hybridization using the myBaits Expert COS Compositae 1Kv1 kit (Daicel Arbor Biosciences, Ann Arbor, MI, USA). For each sample, half of the volume of beads in the elution buffer were PCR-amplified for 10 cycles. Sequencing pools were prepared by pooling the enriched libraries with the unenriched libraries at a 60:40 ratio. The final library pools were quantified again using a spectrofluorimetric assay and a quantitative PCR assay. The samples were sequenced on the Illumina NovaSeq 6000 platform on partial S4 PE150 lanes.

### 2.3. Molecular Data Processing and Phylogenetic Analyses

Given the paleopolyploid origin of the Compositae [53,54] and the evidence of allopolyploidization in the origin of part of the HAP clade [55], accounting for paralogy was key for the analysis of nuclear loci. Accordingly, we used ParalogWizard [34], available at https://github.com/rufimov/ParalogWizard (accessed on 22 February 2022), a workflow that separates orthologs and paralogs of a given locus into two subgroups based on sequence similarity and creates orthologous alignments for each subgroup, which can then be used for phylogenetic tree inference (ParalogWizard is hereafter referred to as PW followed by the number of the respective script). For phylogenetic inference, we used the HybPhyloMaker workflow [56] (available at https://githubp.com/tomas-fer/HybPhyloMaker (accessed on 18 November 2021), indicated hereafter as HPM followed by the number of the respective script).

As a pre-processing step, we removed the adaptors and low quality reads, trimmed the raw reads using Trimmomatic v.0.39 [57], and removed duplicates using BBMap v.38.42 [58] applying HPM1 (reads recovered per species can be found in Table S2). The Compositae1061 probe set [33] was used to generate the reference file for initial read mapping by selecting all sunflower sequence representatives of the targeted loci. Contigs were obtained performing a *de novo* assembly of the reads previously mapped to targets (PW1a and 1b). In order to increase mapping specificity, we then generated a customized reference file based on the best matching longest exonic sequences from our ingroup samples (PW2b). A second round of ParalogWizard was conducted, with paralogy detection turned on, and contigs were assembled to this *Helichrysum*-customized reference (PW1a and 1b). To identify paralogous sequences, pairwise exonic sequence divergence was calculated, resulting in two peaks in the resulting histogram (PW2a). The first peak represents putative allelic variation (exonic contigs with low divergence), while the second peak represents highly divergent exonic contigs, presumably originated from duplication events. Paralogous loci were then separated into two different alignments, based on a reference where the two copies of a paralogous locus were separated (PW2b). The retrieved exonic contigs were matched to this “paralog-prone” reference using BLAT v.34 [59], and the resulting BLAT hits were filtered (for details see Ufimov et al., 2022 [34]). Orthologous alignments were generated using MAFFT v.7.475 [60], and exons were concatenated to loci (PW3). We excluded sequences missing more than 70% data for the locus, and we removed loci for which less than 75% of all samples were represented (HPM5).

Given the ongoing debate on whether concatenation or coalescence-based approaches yield more accurate phylogenetic trees (see Lozano-Fernández 2022 [61]), we followed the standard practice of exploring both. First, we concatenated all loci in a single supermatrix and estimated the best nucleotide substitution model for each locus with ModelTest-NG [62]. Phylogenetic inference under maximum likelihood (ML) was conducted with ten independent tree searches using RAxML-NG v.1.0.3 [63] (modified HPM8f), specifying the best-fit model for each partition and starting from five random and five parsimony-based trees. We assessed branch support through 100 bootstrap replicates and drew support values on the best-scoring ML tree using both Felsenstein’s bootstrap support (BS) [64] and transfer bootstrap expectation (TBE) [65]. Clades with BS and TBE ≥ 70% were considered to be supported. For the summary-coalescent approach, individual gene trees were estimated using RAxML v.8.212 [66] with the general-time-reversible (GTR) substitution model with a gamma distributed rate variation among sites “GTRGAMMA” (following the recommendations [67]) and 500 bootstrap replicates (HPM6a). Based on the best ML tree of these gene trees, a species tree was generated using ASTRAL III v.5.7.8 [68] (HPM7 and HPM8a). Support values were computed as local posterior probabilities (LPP) based on gene tree quartet frequencies [69]. Branches with LPP ≥ 0.95 were considered to be supported. Resulting trees were visualized in FigTree v.1.4.3 [70].

### 2.4. Divergence Time Estimation

We used the penalized likelihood method [71] implemented in the software treePL v.1.0 [72] to time-calibrate the highest-likelihood phylogenetic tree. This approach is particularly suited for large genomic datasets [72]. Three calibration points (CP) in total were used as node age constraints. The lack of relatively old fossils from the Gnaphalieae makes it impossible to include any primary calibration point based on fossil evidence. Two secondary CPs were extracted from Nie et al. (2015) [36]: the HAP clade crown node (CP1) 15.4 million years ago (Mya) and the *Anaphalis* + Mediterranean-Asiatic *Helichrysum* crown node (CP2) 7.04 Mya. Both CP1 and CP2 were used as fixed ages. Although for secondary calibration points it would be preferable to apply a normal or lognormal distribution [73,74], this is not possible in treePL. CP3 was a primary calibration point based on geological evidence, corresponding to the emergence of the archipelago of Madeira 5.6 Mya [75], which was applied as a maximum age at the stem node of the clade grouping the four Madeiran endemic *Helichrysum* species [24]. The dating process consisted of an initial run to detect the optimal parameter settings (opt = 4, optad = 2, and optcvad = 1). We ran a second analysis, replicating the random cross-validation procedure to identify the best smoothing value (allowing it to vary from 0.001 to 1000) using a chi-squared test. Finally, the time-calibrated tree was obtained applying the optimal smoothing parameter (0.01).

### 2.5. Ancestral Range Estimation

We collected information on distribution area, altitudinal range, and habitat for each species from available floras, online resources, and databases [16,26,30,38,39,40,41,42,43,44,45,46,47,48,49,50,51,52], as well as from the labels of the studied herbarium vouchers (see Table S1).

**Figure 2:**
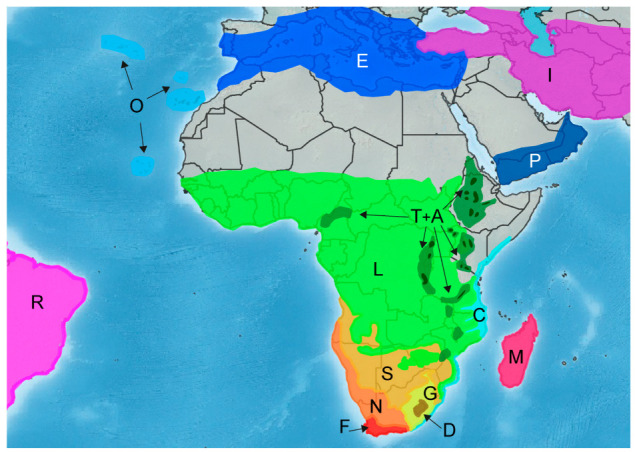
Geographical representation of the 15 geographic areas defined in the current study. These include six areas outside of continental Africa: E: the Mediterranean area; I: Asia; M: Madagascar; O: the Macaronesian area; P: the southern Arabian Peninsula; and R: the Americas. We defined nine areas within continental Africa (from north to south): L: the tropical African lowlands; T: the tropical Afromontane area; A: the tropical Afroalpine area; S: the southern African savannah; N: the arid to semi-arid southern African area; G: the southern African grasslands; D: the high Drakensberg area; C: the Indian Ocean coastal belt forest; and F: the Fynbos biome area. The dark green spots within the tropical Afromontane area are an overrepresentation of the tropical Afroalpine area. The colors and letters correspond to those in the biogeographical reconstruction (Figure 3).

We defined 15 geographic areas reflecting the main distribution patterns of the sampled species (Figure 2). For Africa, areas were mostly based on the natural vegetation regions shown in [8] with small modifications.

First, being aware of the phytogeographical and ecological mismatches of the Afromontane concept exposed in Carbutt and Edwards (2015) [9], we delimited the global Afromontane area following the criteria of Gehrke and Linder (2014) [4], adding some massifs (Chimanimani, Mount Cameroon, and part of the Ethiopian highlands). However, for the biogeographic analysis, we subdivided it into five smaller geographical areas (from north to south): (P) the mountains of the southern Arabian peninsula; (T) the tropical Afromontane area, for species found between 1800 and 3200 m.a.s.l. in the intertropical zone; (A) the tropical Afroalpine area, for species mostly found above 3200 m.a.s.l. or, if they grow below this limit, whose upper altitudinal limit is above 4500 m.a.s.l. in the intertropical zone; (G) the southern African grasslands (below the tropic of Capricorn), including the Drakensberg montane belt, locally known as the “highveld” grasslands, for species mostly growing between 900 and 2600 m.a.s.l.; (D) the high Drakensberg area, for species whose lower altitudinal limit is above 1800 m.a.s.l. and their upper altitudinal limit is above 2600 m.a.s.l. Therefore, our high Drakensberg area is equivalent to the Drakensberg Mountain Centre (DMC) [9,29], which comprises the upper montane belt (above 1800 m.a.s.l., previously considered the subalpine belt [76,77]) and the Drakensberg Alpine Center (DAC, above 2800 m.a.s.l. [76,77]). In this work, we considered Madagascar as a whole unique independent area (M) due to computational limitations of BioGeoBEARS, despite the fact that the highest mountainous areas of Madagascar are generally considered to be part of the Afromontane area [4]. Additionally, non-mountainous areas within continental Africa were defined as follows, from north to south: (L) the tropical African lowlands, for species mostly growing below 1800 m.a.s.l. in the intertropical zone; (S) the southern African savannah; (N) the arid to semi-arid southern African area, which includes the Namib Desert, Succulent Karoo, and Nama Karoo; (C) the Indian Ocean coastal belt forest area; and (F) the Fynbos biome area. To precisely delimit the proposed southern African areas, we checked the maps included in [76,77]. Finally, we defined more broadly four additional areas outside continental Africa: (E) the Mediterranean area; (I) Asia; (O) the Macaronesian area; and (R) the Americas.

Species occurrence in the geographical areas was assigned considering habitat information in combination with distribution and altitudinal range. Some species are only occasionally or marginally present in other areas outside of their main distribution range. In these cases, we only considered the core area(s) or altitudinal belt in which there are a significant number of occurrences of the species, or that can be interpreted as the central distribution area of the species.

We estimated geographic range evolution with the R package BioGeoBEARS v.1.1.2 (available at https://github.com/nmatzke/BioGeoBEARS (accessed on 25 August 2022)) [78] using as input the time-calibrated tree after pruning the outgroup. The maximum number of areas for any node was set to three, which is the highest number of areas occupied by the most widespread extant taxon in our study. We tested the fit of three biogeographical models: the dispersal–extinction–cladogenesis model (DEC) [79,80]; a likelihood implementation of the dispersal–vicariance analysis model (DIVA, hereafter DIVAlike) [81]; and BAYAREAlike [82], a model that decouples range evolution from cladogenesis. In addition, a more complex version of each of the mentioned models was tested through the addition of the jump-dispersal parameter j (DEC+j, DIVAlike+j, BAYAREAlike+j), which depicts founder-event speciation (range switch that occurs at a lineage-splitting event, resulting in one daughter lineage in a new range and the other daughter lineage retaining the ancestral range [83]). We assessed model fit with the Akaike Information Criterion (AIC) and AIC weights (AICwt). We also conducted a Biogeographic Stochastic Mapping (BSM) analysis [84] with 100 replicates to obtain the overall means of anagenetic and cladogenetic events conditional on the geographic distributions, the phylogeny, and the best-fitting model.

## 3. Results

### 3.1. Alignment Processing and Filtering

For each locus, we considered sequence divergence values between 7.6% and 18.9% to indicate paralogy (Figure S1). Using the *Helichrysum*-customized reference, 225 paralogous loci were detected on average (Table S3), with little variation among taxa (sd ± 3.4). We recovered 927 loci, of which 833 were retained and used to build orthologous alignments after filtering for missing data (Table S4). Alignment length for each locus averaged 318 bp (range 49–816 bp; Table S5). On average, each alignment had 116 variable sites and 73 parsimony informative sites.

### 3.2. Phylogenetic Analyses

Concatenation (RAxML-NG; Figure S2) and summary-coalescent (ASTRAL; Figure S3) approaches yielded species trees with similar topologies, especially for the deepest and shallowest nodes. Deep nodes were well supported in the concatenation-based analyses (BS and TBE metrics), but less supported in the summary-coalescent tree. Most of the shallow nodes were strongly supported by all metrics (in both the concatenation and summary-coalescent analyses). In sum, 79% of the nodes were supported with BS ≥ 70%, and 69% of these nodes received 100% BS support; 90.2% of the nodes were TBE supported, and 60.7% of these nodes received a TBE support of 1. In the phylogeny obtained using ASTRAL, 59% of the nodes were supported (LPP ≥ 0.95), and 82.2% of these nodes obtained the maximum support (LPP = 1).

### 3.3. Divergence Time and Ancestral Range Estimation

**Figure 3:**
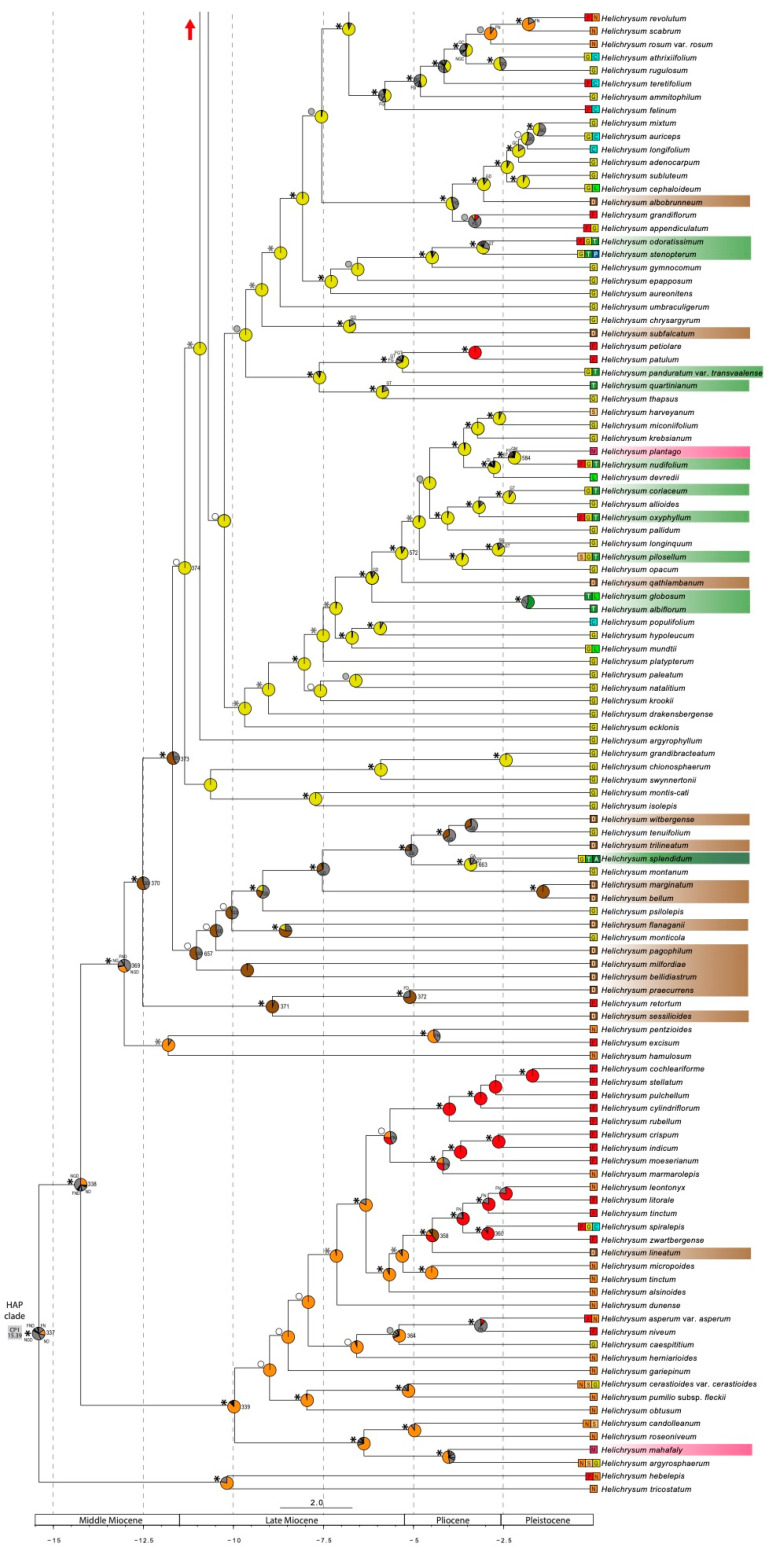
Ancestral range estimation of the HAP clade using the best-fitting model DEC+j based on a time-calibrated phylogeny generated under the concatenation approach using target-enrichment data (Compositae1061 probe set). Pie charts at nodes show the relative probability of the possible states (areas in primary colors; combinations of areas in grey). Relevant node numbers are indicated to the left of the node. Black asterisks indicate nodes that were strongly supported by both metrics (BS and TBE ≥ 95%/0.95), grey asterisks indicate nodes strongly supported by only one metric (BS or TBE ≥ 95%/0.95), grey circles indicate nodes moderately supported by both metrics (BS and TBE from 70%/0.70 to 94%/0.94), and empty circles indicate nodes moderately supported by only one metric (BS or TBE from 70%/0.70 to 94%/0.94). Highlighted species correspond to tropical Afromontane clades in green, which include tropical Afroalpine clades in dark green (labelled as TA1, TA2, TA3); high Drakensberg species in brown; Malagasy species in magenta (main clades labelled as MAD1, MAD2, MAD3, MAD4). Other genera and large Malagasy clades are collapsed. The complete tree obtained in this analysis is shown in Figure S2.

The treePL analysis estimated that the HAP clade originated 15.4 Mya and started to diversify 14.2 Mya (Figure 3 and Figure S2; the former figure is a partially collapsed phylogenetic tree to make it easier to read, Figure S2 is the complete tree). In the biogeographic analysis, DEC+j was the best fitting model according to AIC (AICc > 2 points lower than the second-best model; AICcwt = 1). Founder–event speciation processes (j = 0.0065) had a larger contribution than range expansion by dispersal (d = 0.0027) and range contraction (e = 1 × 10^−12^) (Table S6, Figure S2). In the last years, there has been a controversy regarding the inclusion of the founder-event parameter (+j) in event-based models. Specifically, Ree and Sanmartín (2018) [85] claimed that the comparison of DEC and DEC+j models is statistically invalid, based on two small hand-constructed datasets. Recently, Matzke (2022) [86] presented a thorough simulation-based study that validates the log-likelihood comparison of DEC and DEC+j. In our case, the results based on the DEC model provided in some cases unrealistic range estimations (Figures S5 and S6, Table S9): for instance, the ancestor of the clade which includes numerous Malagasy species (from *H. stilpnocephalum* to *H. chamaeyucca,* MAD1 and MAD2 in Figure 3) and only one Afromontane species (*H. brunioides*), is inferred to have a distribution in the Afromontane area + Madagascar. Another example concerns the ancestor of *Pseudognaphalium* + *Achyrocline*, which is inferred to have a distribution in the Afromontane area + America in the DEC model (whereas the DEC+j model infers only America as the ancestral range). These two cases, among others, exemplify the relevance of the +j parameter in a study case as the one presented here, in which many disjunct distributions are much more probably originated by dispersion and founder event given the recent age of the studied group and the long-term isolation of some of the territories: the separation of America from Africa dates from around 100 Mya [87] and the separation of Madagascar from Africa dates from 165 Mya onwards [88]. In a similar way, most of the current Afromontane areas have been isolated for a long time, and Afroalpine areas from different massifs have never been in contact. For all these reasons, we only discuss the results obtained under the DEC+j model, the best-fitting model for our data according to AICc.

Based on the inferred ancestral range of the basal nodes (probabilities in Table S7), the origin and initial diversification of the genus took place in the arid to semi-arid southern Africa area (N), possibly involving one or more other areas (the southern African grasslands, G; the Indian Ocean coastal belt forest, C; the high Drakensberg area, D; and/or the Fynbos biome, F). The basal-most node separated a clade of two arid southern African taxa (N) from the remaining taxa, which formed two main lineages (Figure 3). The smallest lineage (node 339, BS = 100%, TBE = 1) diversified within the ancestral area (N) but also spread to the Fynbos area (F) around 5.65 Mya (BS = 57%, TBE = 0.785). The largest lineage (node 369, BS = 100%, TBE = 1) split into two clades approximately 13 Mya, a small one that remained in western South Africa, and another (node 370, BS = 98%, TBE = 0.99) that diversified in the high Drakensberg area and the southern African grasslands. Diversification in the southern African grasslands included multiple dispersals towards the east and the north that took place in different time periods, comprising *Helichrysum* species from all geographic areas, including Madagascar and other continents. In this clade, the analyses also recovered back-colonizations from Madagascar to mainland Africa, as well as dispersals from the tropical Afromontane area to the surrounding arid tropical African lowlands.

**Figure 4:**
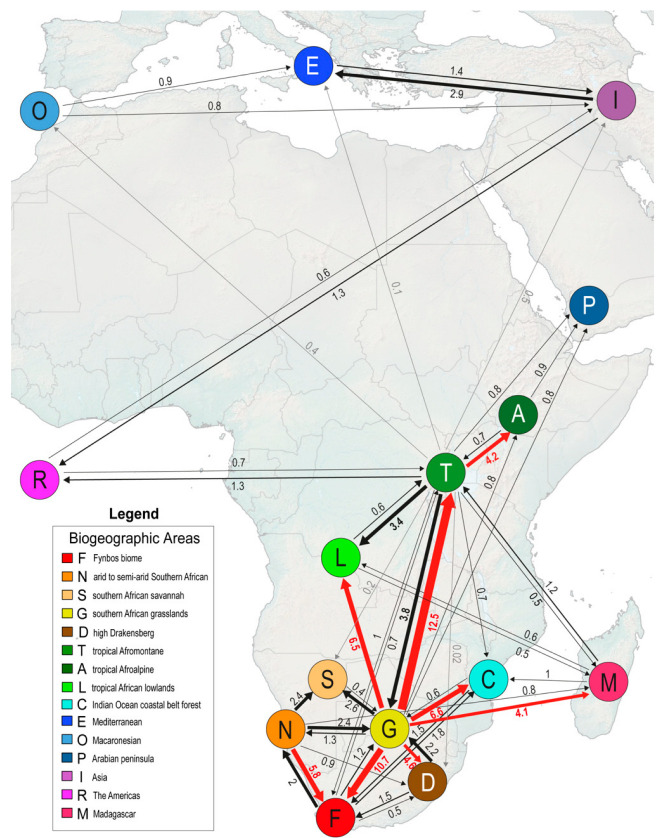
Summary of mean dispersal events estimated from 100 biogeographic stochastic mappings in *Helichrysum* (see all event counts in Table S8). Arrow tips indicate the directionality of the dispersals. Numbers on the arrows are the mean of dispersal event counts. Arrow thickness is proportional to the mean number of dispersals: gray arrows correspond to mean counts below 0.5 (for readability, only the ones involving the eastern African montane area are shown), black arrows represent mean dispersal events ranging from 0.5 to 4 counts, and red arrows show the most common dispersal pathways (mean above 4 counts).

Biogeographic stochastic mapping (BSM) analyses show that *Helichrysum* reached the tropical Afromontane area at least 13 times, with the mean number of events being 17.77 (Figure 4; BSM frequency distributions in Figure S4; BSM summaries in Table S8), mainly from the southern African grasslands (G). This number included colonization events that resulted in the origin of new tropical Afromontane endemic species and range expansions of widespread species. Most of the tropical Afromontane species were found in three lineages (Figure 3). The largest lineage (TA1) started to diverge 9.9 Mya (node 378, unsupported) and gave rise to new lineages that colonized regions outside Africa (mainly Eurasia, the Americas, and Macaronesia). The TA1 lineage diversified in the tropical Afromontane area as well (node 392, BS = 88%, TBE = 0.9), reached the tropical Afroalpine area twice independently (nodes 407 and 412, both with BS = 100%, TBE = 1), and returned twice to the southern African grasslands (nodes 394 and 419, both with BS = 100%, TBE = 1). Another tropical Afromontane lineage (TA2) originated 8 Mya (node = 514, BS = 100%, TBE = 1); it included several tropical Afroalpine species in two lineages and one species from the Arabian Peninsula. The third tropical Afromontane lineage (TA3) originated 3.13 Mya (node = 610, BS = 80%, TBE = 0.9) and gave rise to the tropical Afroalpine *H. forskahlii* var. *compactum* (Vatke) Mesfin. Some tropical Afromontane species occurred scattered across the tree (e. g. *H. quartinianum* A. Rich, *H. albiflorum* Moeser) and mainly originated from southern African grasslands ancestors. Additional colonizations of the tropical Afromontane area took place recently, exemplified by species with a large distribution area extending from South Africa to the tropical Afromontane area (e.g., *H. stenopterum* DC., *H. odoratissimum* Sweet, *H. oxyphyllum* DC., *H. coriaceum* Harv., *H. pilosellum* Less., *H. lepidissimum* S. Moore, *H. acutatum* DC.).

*Helichrysum* reached the tropical Afroalpine area at least four times from tropical Afromontane ancestors. One colonization led to two tropical Afroalpine lineages found in clade TA1: one with *H. amblyphyllum* Mattf. and *H. stuhlmannii* O. Hoffm. (node 412, BS = 100%, TBE = 1), which split 2.3 Mya, and one with *H. newii, H. chionoides* Philipson and *H. brownei* S. Moore (node 407, BS = 100%, TBE = 1), which split 1.9 Mya. A third tropical Afroalpine lineage was nested within TA2 and comprised the Ethiopian species *H. gofense* Cufod. and *H. citrispinum* Delile. A fourth tropical Afroalpine lineage was only represented by the Ethiopian *H. horridum* Sch. Bip., also embedded in clade TA2 and sister to the Arabian species *H. arwae* J.R.I. Wood; this lineage diversified 5.3 Mya (node 515, BS = 100%, TBE = 1). *Helichrysum forskahlii* var. *compactum* (TA3) represented the fourth colonization of the tropical Afroalpine area from a tropical Afromontane ancestor. Lastly, *H. splendidum* has a wide disjunct distribution area in the mountains from the southern African grasslands to the tropical Afroalpine area.

Notably, the tropical Afroalpine species were not found to be closely related to the alpine taxa in the high Drakensberg area (D). Some of the high Drakensberg species were resolved in a lineage (node 657, BS = 24%, TBE = 0.924) that started to diversify 11 Mya and also included southern African grasslands species. Another lineage included two high Drakensberg species (*H. sessilioides* Hilliard and *H. praecurrens* Hilliard) plus the Fynbos species *H. retortum* Willd. (node 372, BS = 100%, TBE = 1). Five other high Drakensberg species were found scattered across the tree (*H. albobrunneum* S. Moore, *H. subfalcatum* Hilliard, *H. qathlambanum* Hilliard, *H. confertum* N. E. Br., and *H. lineatum* Bolus). Four of them originated from ancestors in the surrounding grasslands, whereas *H. lineatum* probably diverged from a Fynbos (F) ancestor 4.5 Mya (node 358, BS = 100%, TBE = 1). In the high Drakensberg area, most speciation events took place between 11 and 1.4 Mya.

*Helichrysum* colonized Madagascar at least six times. The oldest Malagasy (MAD1 + MAD 2) lineage dispersed there from Africa ca. 7.9 Mya (node 522, BS = 100%, TBE = 1). The youngest Malagasy lineage (MAD4, node 613, BS = 100%, TBE = 1) dispersed from Africa around 3.7 Mya and notably diversified in Madagascar 2.2 Mya.

### 3.4. Number, Type, and Directionality Estimation of Biogeographical Events

We estimated from a summary of 100 BSM replicates that within-area speciation events predominated in *Helichrysum* (62.8% of speciation events), followed by dispersal events leading to diversification (28.4%), whereas vicariant events were rare (Table 1). Within-area speciation events appeared to have occurred mainly in the most species-rich areas, the southern African grasslands (G, 32%), and Madagascar (M, 23.5%). Founder events (12.5%) were almost as frequent in the history of *Helichrysum* as range expansions (15.9%).

The relative importance of an area as source or sink for dispersals was similar, independently of the type of dispersal (range expansion vs. founder events). The most frequent dispersals occurred from the southern African grasslands (G) towards the adjacent areas, with the Fynbos biome (F) and the tropical Afromontane area (T) being the most common sinks. Madagascar (M) was the only area where lineages appeared to have originated mainly by founder event speciation rather than range expansion, and these founder events seemed to have originated mainly from southern African grassland ancestors. Movements between the tropical Afromontane area (T) and the southern African grasslands (G) were highly asymmetric, with migrations from G to T being four times more common than from T to G. The high Drakensberg species (D) seemed to derive mainly via colonization from the surrounding grasslands and were never inferred to have spread further north. All tropical Afroalpine species descended from tropical Afromontane ancestors.

## 4. Discussion

### 4.1. Utility of Target-Enrichment Strategies in Reconstructing the Radiation of Helichrysum

Resolving relationships of young rapidly radiating groups is a well-known difficult task in the field of plant systematics. Researchers have to deal with high levels of tree discordance, consequences of evolutionary processes (i.e., ILS, hybridization, whole-genome duplication), and methodological artifacts. Hyb-Seq and related target-enrichment approaches have proven to be efficient in generating genomic data to deal with complex groups [89]. Recent studies, aware of the gap between advances in sequencing techniques and data analysis approaches to deal with such incongruences, suggest some guidelines to yield more accurate phylogenomic inferences and improve our understanding of evolution (e.g., *Burmeistera* H. Karst. & Triana [90]; *Dendrosenecio* [91]; Lobelioideae [92]; and *Loricaria* Wedd. [93]). The target-enrichment dataset generated here, coupled with a recent pipeline that detects and uses paralogs for phylogenetic reconstruction (ParalogWizard [34]), allowed us to build the first highly resolved phylogeny of the HAP clade. This phylogeny is based on 833 loci and wide taxonomic sampling (304 species, representing ≈50% of the diversity in *Helichrysum*, and 31 species from smaller, closely related genera). Since the taxonomic sampling is not yet complete, we cannot discard that increased taxon sampling may affect phylogenetic inference and biogeographic estimation; however, to minimize this potential bias, our current sampling was designed to evenly represent all major taxonomical and biogeographical groups and was highly increased in relation to previous works. The most comprehensive nuclear phylogeny of *Helichrysum* previously published included only 145 species, which represented ≈25% of the diversity in *Helichrysum* and was based on nrDNA ITS and ETS markers [24]. Despite the different sampling, a similar general pattern has been recovered: (1) the early divergent lineages of *Helichrysum* are from SW Africa; (2) the Afromontane and Afroalpine species appear in several independent clades, i.e., they have multiple independent origins; (3) the Malagasy species appear in several clades, i.e., they have multiple independent origins; (4) the genera *Achyrocline*, *Anaphalis*, *Pseudognaphalium*, and *Humeocline* are nested in the main HAP clade; (5) the genus *Anaphalis* is sister to the Mediterranean–Asiatic–Macaronesian *Helichrysum*. However, the statistical branch supports are notably higher in the present approach, and some differences have emerged in the relationships obtained, among which we highlight the following: (1) although the early divergent lineages are from SW Africa in both phylogenies, they are grouped differently, i.e., they constituted several small clades placed in a grade at the base of the previous phylogenies; (2) the sister lineages of the Afromontane species were not resolved or statistically supported in the previous study, whereas in the current work they are; (3) the genus *Pseudognaphalium* constituted a unique clade in previous phylogenetic studies, whereas in the present one the species of this genus are distributed in two main clades, one of which is grouped with *Achyrocline*, and the other with *Anaphalis* and the Mediterranean–Asian–Macaronesian species of *Helichrysum*.

The topologies obtained with the concatenation approach (RAxML-NG tree; Figure S2) and the summary-coalescent approach (ASTRAL tree; Figure S3) are largely congruent, especially for the deepest and shallowest nodes. The main topological differences involve intermediate nodes with ages 12–8 Mya, but these are not or only weakly supported in both topologies and have very short branches, suggesting rapid diversification events that are difficult to disentangle [94]. Therefore, the divergence times and biogeographic estimations obtained for these nodes should be taken with caution. These events seem to have occurred during the major uplift of the East African Rift system and the subsequent aridification of eastern Africa in the late Miocene [95]. Successive rapid speciation events are often characterized by strong ILS signatures at many loci [96], which along with hybridization reduce phylogenetic signals. This effect magnifies with genome-scale datasets [97]. Moreover, the Malagasy clades MAD2 (node 523) and MAD4 (node 613) appear to have undergone rapid, but more recent (<2 Mya) diversification, resulting in incongruences (Figures S2 and S3). Notably, the relationships between the tropical Afromontane and tropical Afroalpine species are virtually identical in both phylogenies.

### 4.2. The Early History of Helichrysum and Colonization of Madagascar

Our analyses place the ancestor of the HAP clade in the arid to semi-arid southern African areas during the mid Miocene (ca. 15 Mya; Figure 3). This dating coincides with the beginning of a global cooling trend [98] and the onset of more arid climates in the western parts of southern Africa. The dry summers likely contributed to a notable floristic change from the former subtropical humid forests and woody grasslands [99,100]. Previous studies inferred the Greater Cape Floristic Region (constituted by the Fynbos biome and the Succulent Karoo biome) as the center of origin of the HAP clade [31]. Our new result arises because we added two newly sequenced species from the arid areas of western southern Africa that appear to be at the base of the group, and also because we delimited the biogeographic areas differently, uniting the arid winter-rainfall Succulent Karoo with the arid summer-rainfall regions of southern Africa (Nama Karoo, Namib desert) and not with the Fynbos as the former study did. However, there is no discrepancy between the two reconstructions if the northern part of the Succulent Karoo (“Namaqualand”) is considered as the ancestral area for the HAP clade.

One of the two main *Helichrysum* clades first diversified in the arid areas of western southern Africa and subsequently colonized and diversified in the adjacent Fynbos biome. In contrast, the second main clade dispersed rapidly eastwards in South Africa in the middle to late Miocene, colonizing the southern African grasslands (G) and the high Drakensberg area (D). Several additional colonizations of these grasslands from arid-adapted ancestors occurred more recently. The colonizations of both the southern African grasslands and the high Drakensberg were followed by in situ diversifications, likely fostered by the opening of new habitats following the Miocene–Pliocene Drakensberg uplift [41,101,102,103] and coinciding with many other major radiations during that time (see Hughes and Atchison 2015 [104] for a global review on montane and alpine plant groups; also in other organisms such as rodents [105] and butterflies [106]). The biogeographic connection of southwestern Africa and the eastern African mountain systems is not surprising because even today, the arid southern African areas experience local frosts [107], and this was likely more severe during past times, meaning that previous *Helichrysum* lineages could already be tolerant to cold conditions, which potentially would have facilitated the colonization of mountain habitats, characterized by cold climatic conditions.

*Helichrysum*’s grasslands ancestors also dispersed multiple times to adjacent southern African areas, lowland tropical Africa, and Madagascar during the late Miocene to Early Pliocene. Dispersals from the southern African grasslands to other areas were often followed by in situ diversification and in some cases by back-colonization events, suggesting a highly dynamic biogeographic history for *Helichrysum* (Figure 4, Table S8).

Our study also provides new insights into the biogeographic history of the Malagasy species. A previous study only included 18 species from Madagascar and was based on few DNA markers, resulting in poorly resolved phylogenies [24]. Here, using many markers and including 65 Malagasy species (57% of the island’s species), we identified at least six independent dispersal events from mainland Africa to Madagascar, with the southern African grasslands as the main source area (Figure 4). The first colonization of Madagascar is inferred at around 7.9 Mya, and the most recent one around 3.7 Mya. Since Madagascar was well separated from the African continent already in the early Cretaceous [88], all colonizations by *Helichrysum* probably occurred via wind- or water-mediated LDD across the Mozambique Channel [108]. Most Malagasy *Helichrysum* colonized the cool temperate highlands. However, *H. mahafaly* colonized Madagascar around 4 Mya from the arid southwestern African area. It is the only Malagasy species occurring in the arid southwestern lowlands, demonstrating niche conservatism. Notably, we identified two back-colonizations from Madagascar to mainland Africa around 5.5 Mya, giving rise to *H. brunioides* Moeser in the tropical Afromontane area and *H. silvaticum* Hilliard in the forests of the southern African east coast.

### 4.3. Repeatedly Northwards

The tropical Afromontane area has been colonized independently at least 12 times from the southern African grasslands (Figure 4, Table S8), and at least once from Madagascar (*H. brunioides*, node 523). Thus, our results support the hypothesis that the southern African grasslands area harbored a fruitful floristic radiation that directly fed the tropical Afromontane flora [109] and played an important role as a transitional environment [25,29]. This biogeographical connection from the Cape region northwards, in which the southern African grasslands act as a stepping-stone area [16,17,18,19], has been reported for other plant groups, such as *Disa* Bergius (Orchidaceae) and the tribe Irideae (Iridaceae), with species numbers decreasing west- and northwards on the African continent as a general pattern [18], as found in *Helichrysum*.

The biogeographic assembly of the tropical Afromontane flora was probably deeply influenced by orogenic events and past climatic changes, as suggested for several plant groups [4]. *Helichrysum* colonized the tropical Afromontane area repeatedly from the late Miocene to the Pleistocene. Some of the high mountains in eastern Africa were already present during the Oligocene [4,95]. However, major uplift events occurred in the Pleistocene, associated with the Great Rift Valley formation [95,110]. Considering the habitat currently occupied by tropical Afromontane *Helichrysum* species (i.e., open forest glades), the climatic cooling trend during the late Miocene [111] and the cold and arid conditions during glacial periods might have fostered a gradual expansion and later isolation of several lineages from the southern African grasslands towards the eastern African mountains.

During cold and dry periods, the extent and altitudinal range of forested areas decreased because the lower altitudinal limit was pushed up by the expanding arid open vegetation, while the expanding glaciers pushed the alpine and ericaceous belts downhill [11]. The increased geographical isolation between the tropical Afromontane areas and the southern African grasslands during unfavorable periods probably fostered the differentiation of tropical Afromontane endemic lineages through allopatric speciation. However, most tropical Afromontane *Helichrysum* species have wide distribution ranges that span several currently isolated mountain massifs, which is consistent with cyclic formation of forest bridges or at least reduced dispersal distances between patches of Afromontane formations during recurrent interglacials [11]. In a few cases, expansion from southern Africa appears to have been more recent, since several species have wide distribution ranges that include both the southern African grasslands and the tropical Afromontane area. For instance, *H. splendidum* (node 663, 3.42 Mya) is nested within the main alpine-adapted high Drakensberg lineage and occurs in the southern African grasslands and tropical Afromontane and Afroalpine areas, and *H. nudifolium* (L.) Less. (node 584, 2.22 Mya) occurs in the southern African grasslands as well as in the tropical Afromontane area and the Fynbos biome area.

In sum, the present-day distribution of *Helichrysum* in the tropical Afromontane area is probably a result of the increased topographical complexity during mountain uplift periods, followed by cycles of colonization, speciation, and extinction during the Pleistocene climatic oscillations, including extinction of ancestral transitional populations [14,20]. One of the main characteristics of *Helichrysum* species are their broad niches, potentially hosting the variation needed for rapid adaptation. These features probably confer these species with adaptive versatility that has facilitated repeated environmental switches and geographic movements.

Interestingly, the tropical Afromontane area, apart from being the recipient of multiple immigrant lineages and hosting several in situ radiations, has probably been the main source of extra-African *Helichrysum* lineages. Our results indicate that both the northern hemisphere *Helichrysum* species (the Asian, Mediterranean and Macaronesian clade; node 434, BS = 36%, TBE = 0.985) and the Asian genus *Anaphalis* (node 471, BS = 100%, TBE = 1) descend from tropical Afromontane ancestors. However, as it is clearly visible from the number of connections among areas (Figure 4) that the Saharan and Arabian deserts have been crossed rarely, and always northwards in this plant group. Such result emphasizes the role of these deserts as biogeographic barriers and indicates that the mountains located in them (Hoggar Mountains, Tassili n’Ajjer Range, Tibesti Mountains, among others) did not play a significant role as steppingstones. Other extra-African radiations that possibly were sourced from tropical Afromontane area include one Malagasy group (MAD4, node 613) and the mostly American genera *Pseudognaphalium* and *Achyrocline*. The large genus *Senecio* (Asteraceae, tribe Senecioneae) shares a similar biogeographic historical pattern with *Helichrysum* [23,112], since it also originated in arid areas of western southern Africa and has been hypothesized to have colonized the Palearctic and the Americas multiple times from different ecosystems of the African continent.

### 4.4. Repeatedly Upwards

All tropical Afroalpine clades recovered in our *Helichrysum* tree are inferred to have originated from tropical Afromontane ancestors, not from ancestors preadapted to alpine conditions in Drakensberg or Eurasia. This scenario contrasts with that of most tropical Afroalpine lineages already adapted to cold climates with a common presence of frost and closely related to northern lineages (e.g., *Arabis alpina* Krock. ex. Steud. [113]; *Cardamine* L. [114]; *Ranunculus* [10]; *Carex* [4,10]; *Alchemilla* [115]; *Lychnis* L. [116]; *Anthoxanthum* L. [117]). In contrast to all these examples, the Mediterranean–Asiatic *Helichrysum* species, which are shown to constitute a unique and highly supported clade (this work and Galbany-Casals et al., 2009 [28]) have not fed the Afromontane and Afroalpine floras but the opposite. Our results indicate that colonization of the alpine areas in the Drakensberg as well as in tropical Africa happened several times independently from *Helichrysum* ancestors occurring in the surrounding lower-elevation montane areas. Thus, repeated parallel adaptation from montane to alpine habitats has prevailed in *Helichrysum*. Indeed, most tropical Afroalpine *Helichrysum* species from different clades share particular morphological traits that suggest convergent adaptive evolution (Figure 1a–c): (i) their capitula are larger than in non-alpine species, possibly evolved to attract scarce pollinators; (ii) their capitula are white (instead of yellow, brown, pink, or reddish), the most attractive color for Diptera and Coleoptera, which were the dominating pollinators we observed in the Afroalpine area during the field expeditions; (iii) their dense pilosity, which protects against high U.V. radiation; and (iv) their nyctinastic capitula (i.e., capitula that close during the night), thus protecting the florets from night frost.

Both the high Drakensberg area and the tropical Afroalpine areas are mainly colonization sinks (as reported for *Erica* in the Drakensberg range [118]) because dispersal to other areas has not been detected or is exceptional. However, three of the tropical Afroalpine lineages of *Helichrysum* diversified in situ after colonization, probably by allopatric speciation. The age estimated for tropical Afroalpine species endemic to a single mountain range suggest that they emerged after periods of volcanic mountain-building activity. For instance, *H. brownei* diverged from its sister species approximately 1.9 Mya on Mt. Kenya, which underwent an uplift 2.6 Mya [95,110].

Establishment of *Helichrysum* in Afroalpine areas could only have happened from the time the alpine habitat became available after mountain uplift, estimated from the Miocene onwards [119,120]. The alpine habitat was first available in the Drakensberg in the mid-Miocene after a major uplift period [101,102,103], and the formation of alpine patches accelerated during the Plio-Pleistocene, when the eastern African mountains reached their current altitudes [4]. The establishment of *Helichrysum* in alpine habitats was probably favored by its preference for open and arid habitats rather than dense forests. The repeated shifts in *Helichrysum* from a geographically proximate but non-alpine biome rather than recruitment via LDD from remote but ecologically more similar alpine biomes suggest that adaptation to extreme daily temperature fluctuations, frosty nights, and high insolation may be more straightforward than often thought [20,25].

Long-distance dispersal of *Helichrysum* appears nevertheless to have been important within the tropical Afroalpine area. LDD was probably facilitated during glacial maxima, when the alpine belts were found at lower elevations (up to 1000 m lower than today [21]), diminishing dispersal distances and increasing the total area of the alpine habitat [20]. This allowed for increases in population size, resulting in increased production and exchange of propagules [4,5,95]. The disjunct occurrence of several tropical Afroalpine *Helichrysum* species in mountains in Kenya, Tanzania, Rwanda, and Uganda (e.g., *H. argyranthum*, *H. formosissimum*, *H. newii*) probably results from LDD, given that the alpine belts of most of these mountains have never been connected [11,21]. Given this, it is likely that wind is the most important dispersal vector for *Helichrysum*—although their pappus are soon caducous and do not seem to have a significant role in dispersion, their achenes are tiny and light [36,121,122]. In this regard, our results are in line with many biogeographic studies suggesting that LDD has played a major role in shaping colonization and genetic structuring of tropical Afroalpine plant communities [20]. For instance, the widespread *Erica arborea* Brot. recently colonized the tropical East African mountains, likely from Ethiopia [123]; gene flow has occurred among distant populations of tropical Afroalpine *Alchemilla* species [115]; and intermountain admixture and hybridization have been reported in the recently radiated *Dendrosenecio* genus [91,124].

## 5. Conclusions

The southern African grasslands are for the first time identified as having played a crucial role in the biogeographic history of *Helichrysum*. Our results indicate that these grasslands constitute the most important source of lineages colonizing other African areas, in particular the tropical Afromontane area. Similarly, the tropical Afromontane area spawned extra-African HAP clade radiations, including dispersal events to Eurasia, America, and Madagascar, and back-colonizations to the southern African grasslands. In general, the ability of *Helichrysum* to span broad niches may have favored repeated niche shifts and multiple colonizations of most areas. Range expansions of montane habitats during favorable periods of the Miocene-Pliocene, followed by phases of retraction and habitat isolation between mountain groups, likely fostered diversification and shaped the current Afromontane species diversity. Regarding the origin of the tropical Afroalpine species, our data do not provide evidence for dispersal from other alpine areas. Instead, we detected several independent colonization events of the tropical Afroalpine area from the geographically close tropical Afromontane area by ancestors that were likely preadapted to open habitats, suggesting repeated niche shifts to adapt to the extreme diurnal temperature oscillation and night frost of the Afroalpine area. Downwards elevational expansion of the tropical Afroalpine belt during glacial periods [10,20] likely favored dispersal among Afroalpine “islands” due to the increase in target habitat size [4,10]. The combination of allopatric speciation and range expansion of some species is likely to have shaped the current diversity and distribution of tropical Afroalpine *Helichrysum*. In general, our results agree with studies suggesting that tropical Afroalpine plant radiations were both rapid and recent (Pliocene–Pleistocene) and triggered by the appearance of new ecological opportunities provided by mountain uplift and climatic oscillations [13,98].

## Figures and Tables

**Table 1:** Summary of 100 biogeographic stochastic mapping counts for all *Helichrysum* ranges using the DEC+j model. Mean number of events estimated are shown along with standard deviations (sd). Range-switching dispersals (a) and range contractions (e) are not shown because those parameters were not required in the best fitting model (DEC and related). * Within-area speciation, subset type: the new species occupies a subset of the ancestral range. For more information on the modes, visit http://phylo.wikidot.com/biogeobears (accessed on 5 September 2022).

Mode	Type	Mean (sd)	% of Biogeographic Events
Within-area speciation	Sympatry	250.2 (3.95)	62.8
	Subset *	23.38 (5.2)	5.9
Dispersal	Founder events	49.9 (4.28)	12.5
	Range expansions	63.18 (2.64)	15.9
Vicariance	Vicariance	11.56 (2.85)	2.9
Total		398.2 (2.64)	100

## Data Availability

Raw sequences generated during this study were deposited in the NCBI Short Read Archive database (SRA) under the BioProject accession number PRJNA936872 (access: https://www.ncbi.nlm.nih.gov/sra/PRJNA936872 (accessed on 15 March 2023)). BioSample accession numbers for each sample can be found in the Supplementary Materials along with herbaria codes and voucher information (Table S1).

## Supplementary Materials

The following supporting information can be downloaded at https://www.mdpi.com/article/10.3390/plants12112213/s1. Figure S1: ParalogWizard pairwise distance histogram. Figure S2: Maximum likelihood ancestral range estimation of *Helichrysum* using the best-fit model DEC+j from BioGeoBEARS. Figure S3: ASTRAL species tree of *Helichrysum*. Figure S4: Frequency distributions of event counts from 100 biogeographic stochastic mappings (DEC+j model) on the *Helichrysum* phylogeny. Figure S5: Maximum likelihood ancestral range estimation of *Helichrysum* using the model DEC from BioGeoBARS. Figure S6: Frequency distributions of event counts form 100 biogeographic stochastic mappings (DEC model) on the *Helichrysum* phylogeny. Table S1: List of studied materials with BioSample accession numbers. Table S2: Reads recovered per species after filtering (HPM1). Table S3: Statistics from paralogy analyses using ParalogWizard. Table S4: Summary of selected loci for subsequent phylogeny reconstruction. Table S5: Summary statistics resulting from the processing of nuclear DNA data. Table S6: Summary statistics of biogeographic model testing in BioGeoBEARS. Table S7: Ancestral range probabilities of the model DEC+J for *Helichrysum*. Table S8: DEC+j model biogeographic stochastic mapping mean and standard deviation tables. Table S9: DEC model biogeographic stochastic mapping mean and standard deviation tables.
