# Supplementary material for: Repeatedly Northwards and Upwards: Southern African Grasslands Fuel the Colonization of the African Sky Islands in Helichrysum (Compositae)
Source: Plants (Basel). 2023 Jun 3;12(11):2213. doi: 10.3390/plants12112213 (PMC10255704; doi:10.3390/plants12112213)
Supplement: Supplementary file 1 [file plants-12-02213-s001.zip › Figure S2_Helichrysym_DECj_202305.pdf]

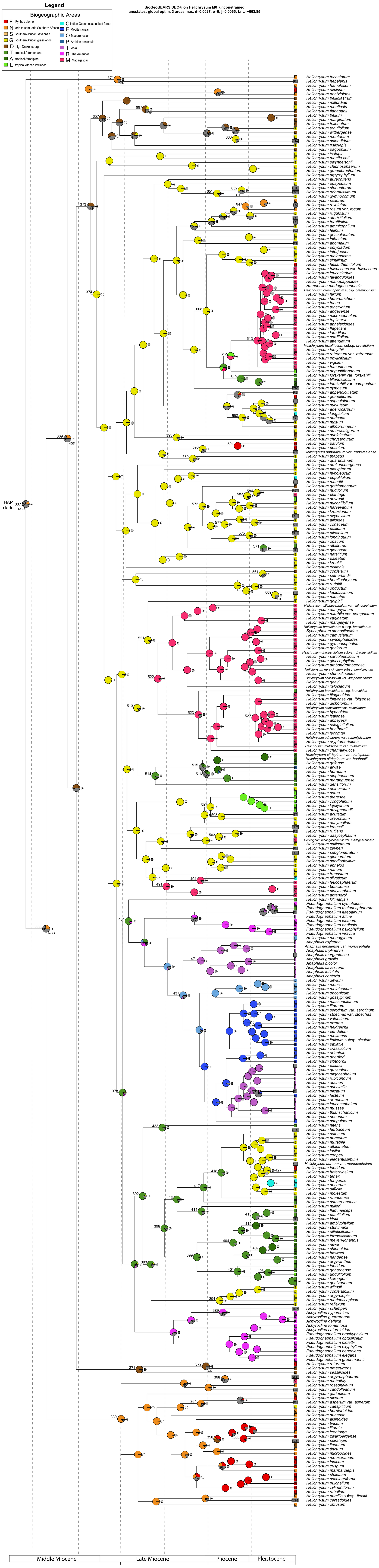

Figure S2. Ancestral range estimation of the HAP clade using the best-fitting model DEC+j based on a time-calibrated phylogeny generated under the coalescence approach using target-enrichment data (Compositae1061 probe set). Pie charts at nodes show the relative probability of the possible states (areas in primary colors; combinations of areas in grey). Relevant node numbers are indicated to the left of the node. Black asterisks indicate nodes that were strongly supported by both metrics (BS and TBE ≥ 95%/0.95), grey asterisks indicate nodes strongly supported by only one metric (BS or TBE ≥ 95%/0.95), grey circles indicate nodes moderately supported by both metrics(BS and TBE from 70%/0.70 to 94%/0.94), and empty circles indicate nodes moderately supported by only one metric (BS or TBE from 70%/0.70 to 94%/0.94).
