# Supplementary material for: Repeatedly Northwards and Upwards: Southern African Grasslands Fuel the Colonization of the African Sky Islands in Helichrysum (Compositae)
Source: Plants (Basel). 2023 Jun 3;12(11):2213. doi: 10.3390/plants12112213 (PMC10255704; doi:10.3390/plants12112213)
Supplement: Supplementary file 1 [file plants-12-02213-s001.zip › Figure S4_DEC+j_histograms_of_event_counts.pdf]

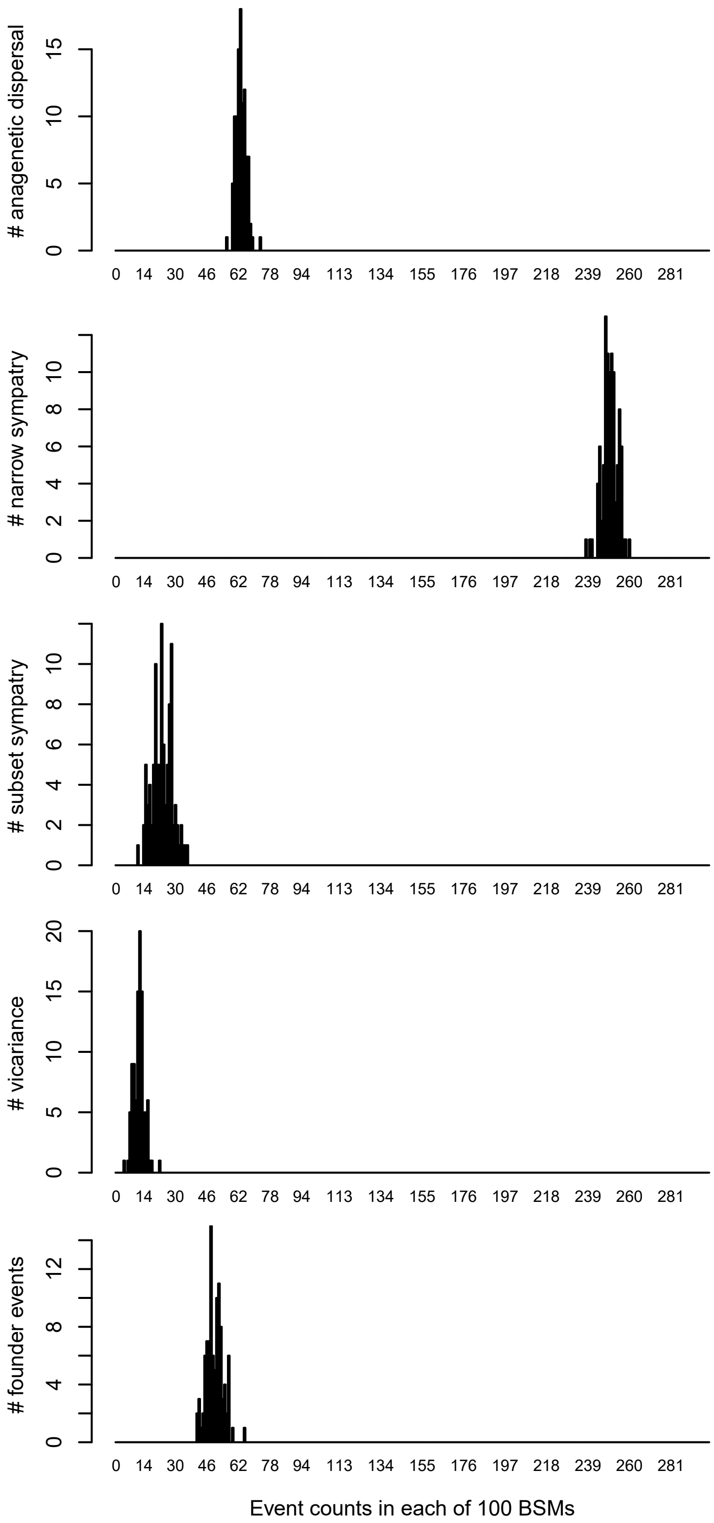

Figure S4. Frequency distributions of event counts from 100 biogeographic stochastic mappings (DEC+j model) on the *Helichrysum* phylogeny.
