# Supplementary material for: Repeatedly Northwards and Upwards: Southern African Grasslands Fuel the Colonization of the African Sky Islands in Helichrysum (Compositae)
Source: Plants (Basel). 2023 Jun 3;12(11):2213. doi: 10.3390/plants12112213 (PMC10255704; doi:10.3390/plants12112213)
Supplement: Supplementary file 1 [file plants-12-02213-s001.zip › Figure S5_Helichrysym_DEC_202305.pdf]

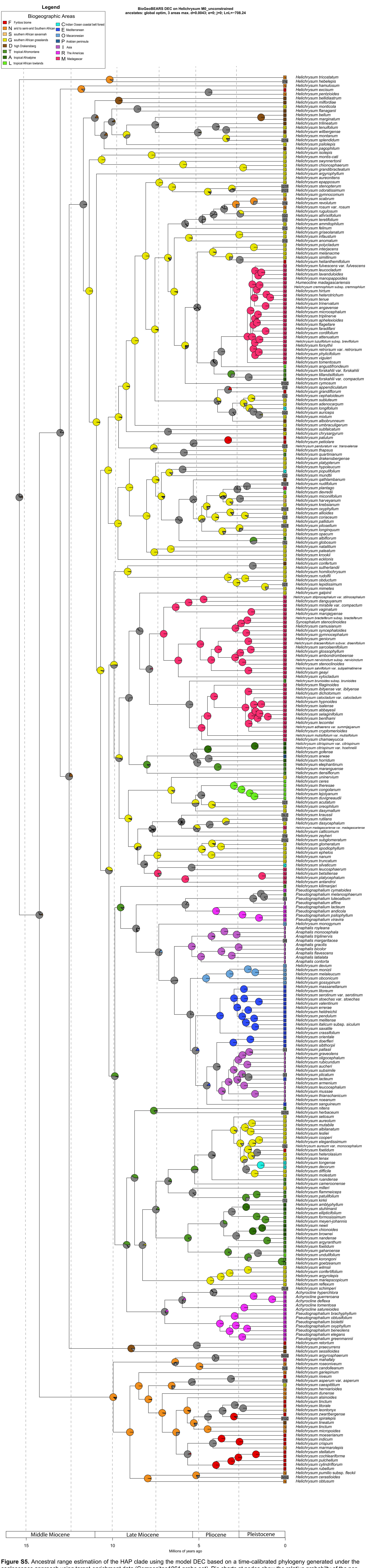

Figure S5. Ancestral range estimatiion of the HAP clade using the model DEC based on a time-calibrated phylogeny generated under the coalescence approach using target-enrichment data (Compositae1061 probe set). Pie charts at nodes show the relative probabilty of the possible states (areas in primary colors, combinations of areas in grey).
