# Supplementary figures and images for: Repeatedly Northwards and Upwards: Southern African Grasslands Fuel the Colonization of the African Sky Islands in Helichrysum (Compositae)
Source: Plants (Basel). 2023 Jun 3;12(11):2213. doi: 10.3390/plants12112213 (PMC10255704; doi:10.3390/plants12112213)

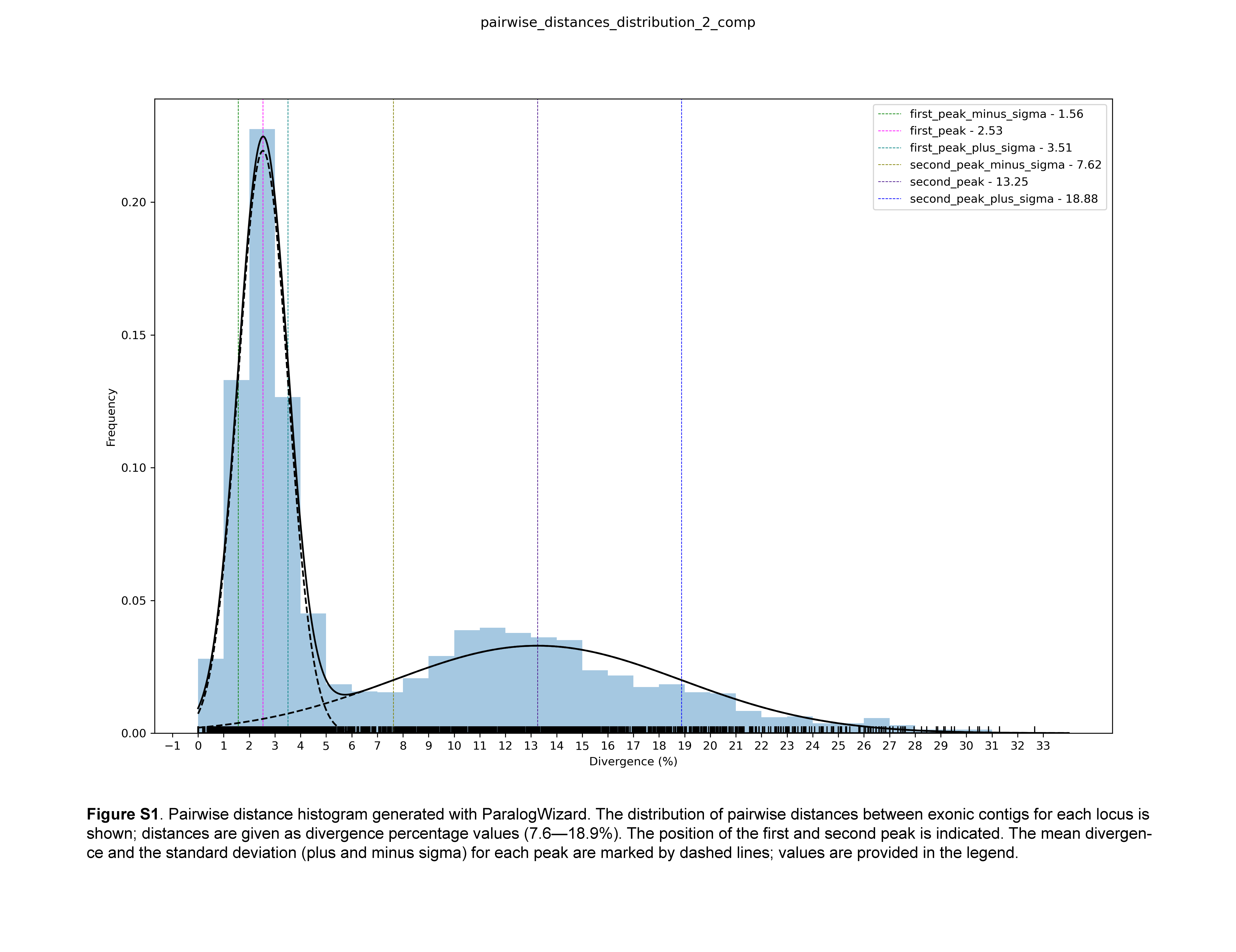

Supplement: Supplementary file 1 [file plants-12-02213-s001.zip › Figure S1_pairwise_distances_distribution_2_comp.tif]

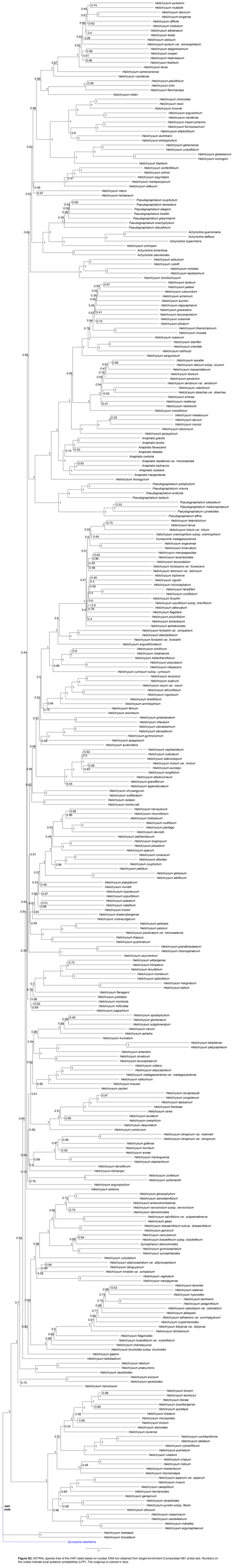

Supplement: Supplementary file 1 [file plants-12-02213-s001.zip › Figure S3_astral_20230508.pdf]
